# Longitudinal associations between loneliness, social isolation, and healthcare utilisation trajectories: a latent growth curve analysis

**DOI:** 10.1007/s00127-024-02639-9

**Published:** 2024-03-01

**Authors:** Qian Gao, Hei Wan Mak, Daisy Fancourt

**Affiliations:** 1 Department of Behavioural Science and Health, Institute of Epidemiology & Health Care, University College London, 1-19 Torrington Place, London, WC1E 7HB UK; 2 School of Public Health, Imperial College London, London, UK

**Keywords:** Loneliness, Social isolation, Health service, Health inequity, Healthy ageing

## Abstract

**Purpose:**

To explore the longitudinal associations between eight-year trajectories of loneliness, social isolation and healthcare utilisation (i.e. inpatient, outpatient, and nursing home care) in US older adults.

**Methods:**

The study used data from the Health and Retirement Study in 2006–2018, which included a nationally representative sample of American adults aged 50 and above (*N* = 6,832). We conducted latent growth curve models to assess the associations between trajectories of loneliness and isolation and healthcare utilisation over 8 years.

**Results:**

Independent of sociodemographic and health-related confounders, social deficits were associated with a lower likelihood of baseline physician visits (loneliness *β*= -0.15, *SE* = 0.08; social isolation *β*= -0.19, *SE* = 0.08), but there was a positive association between loneliness and number of physician visits (*β* = 0.06, *SE* = 0.03), while social isolation was associated with extended hospital (*β* = 0.07, *SE* = 0.04) and nursing home stays (*β* = 0.05, *SE* = 0.02). Longer nursing home stays also predicted better trajectories of loneliness and isolation over time.

**Conclusion:**

Loneliness and social isolation are cross-sectionally related to complex patterns of different types of healthcare. There was no clear evidence that social deficits led to specific trajectories of healthcare utilisation, but nursing home stays may over time help provide social contact, supporting trajectories of isolation and potentially loneliness. Non-clinical services such as social prescribing could have the potential to address unmet social needs and further promote patients’ health-seeking profiles for improving healthcare equity.

**Supplementary Information:**

The online version contains supplementary material available at 10.1007/s00127-024-02639-9.

## Background

Loneliness and social isolation are increasingly recognised to adversely affect health, which may subsequently result in increased health needs and service consumption [1]. Loneliness and social isolation are prevalent in ageing populations: around 1 in 4 older people in the US aged 65 years and older report feeling lonely [2] or socially isolated [3]. Loneliness, a subjective feeling, occurs when a person feels alone despite the amount of social contact, whereas social isolation refers to an objective measure of lacking social connections and interaction. The two concepts are not necessarily concurrent and may exist independently [3]. Both factors are risk factors for incidence and progression of long-term health conditions such as dementia, stroke, frailty and heart failure, leading to an increased demand for healthcare [3, 4]. Further, both are interrelated with other health risk factors, such as socioeconomic status and broader social risk factors such as weaker, narrower and lower quality social relationships [2, 3, 5].

In theory, the mechanisms underlying the relationship between social deficits (such as loneliness and social isolation) and healthcare utilisation can be systematically explained through biological, behavioural, psychological and social pathways [6, 7]. Biologically, there is growing evidence for the associations between social deficits and worsening inflammation, immune and metabolic processes [3, 8, 9]. This biological disruption can increase the risk of the onset and progression of multiple long-term morbidities [9–11], triggering increased demands for intensive healthcare. *Behaviourally*, individuals with social deficits may have greater engagement in unhealthy behaviours (e.g. smoking, poor diet, sedentary behaviours) [12–15] and less interaction with health-promoting activities (e.g. arts, social and cultural engagement) [16]. Both pathways align with the Behavioural Model of Health Services Use [17], which posits that individuals are likely to accumulate ‘needs’ through bio-behavioural pathways, and could support understanding the role of social deficits in influencing healthcare use [18]. *Psychologically*, deficits in social relationships have the potential to influence healthcare utilisation in both directions. As a potential obstacle to accessing healthcare [19], those living with loneliness or social isolation are at greater risk of experiencing stress [9], suicidal ideation, depression [20, 21] and other psychological barriers stemming from lower levels of self-efficacy, poorer expectancies, and more negative beliefs of healthy ageing [22–24], which could fundamentally reduce patient activation and hinder healthcare-seeking [25]. Long-term, ignoring or delaying treatment can worsen health status and involve more specialised and intensive healthcare consumption [26]. But equally, individuals who are lonely or isolated may seek social interaction through increased healthcare utilisation [27], or may have lower health literacy, leading to a lower ability to manage more minor health issues at home [28]. Finally, through *social pathways*, loneliness and social isolation are disproportionately related to more experiences of social disadvantages and life events [3]. Social inequalities such as low social capital, resources and health literacy could directly hinder the healthcare-seeking process and indirectly be translated into biopsychological disparities (e.g. adversely shaping physiologic stress responses), potentially resulting in a deterioration of health and increasing healthcare demands [10].

A number of epidemiological studies have investigated the links between social deficits and healthcare utilisation, providing mixed evidence across service types and study settings. Previous cross-sectional and longitudinal studies have shown that loneliness is associated with increased healthcare utilisation, for example, more frequent primary care visits [29, 30], emergency department visits [30–32], physician visits [33–36] and increased inpatient care, including annual hospitalisation rate [35], emergency hospital admissions [30, 32] and rehospitalisation [34] independent of health confounders. Similarly, social isolation has been reported as a risk factor for hospitalisation among older populations in Australia [37] and the UK [38]. Yet other studies have shown a negative relationship between social deficits and healthcare utilisation. For instance, a recent longitudinal study revealed that individuals living with new or chronic loneliness tended to have fewer physician visits [39]. Social isolation has also been related to reductions in outpatient care use, but with insufficient evidence for GP visits and emergency care [30]. On the other hand, other studies have identified limited or no associations between social deficits and healthcare utilisation, including general or planned inpatient admissions [30, 32, 34, 36], physician visits, hospitalisation and community-based services in older age [40]. Thus, overall, the healthcare utilisation patterns by social isolation or loneliness vary depending on the different measures adopted, service types, and study settings, leading to a need for further exploration.

Consequently, research gaps remain in understanding the longitudinal associations between social deficits and healthcare use. To date, much of the evidence is derived from cross-sectional studies, while some longitudinal investigations are somewhat less representative and transferable due to involving small or specific study samples or short follow-up periods [34, 38]. Both social deficits and healthcare use are likely to fluctuate over time, accompanied by potential interactions between loneliness and social isolation. So there is a need to provide in-depth explorations of the associations between trajectories of loneliness and social isolation and trajectories of healthcare utilisation that can take account of potential bidirectional effects. Moreover, most previous investigations only explored longitudinal relationships between social deficits and limited types of services. Thus, applying an outcome-wide approach by including a wide range of healthcare types in modelling could provide crucial new evidence. In light of this, our study aimed to explore the longitudinal associations between eight-year trajectories of loneliness, social isolation and healthcare utilisation (i.e. inpatient, outpatient, and nursing home care) using a nationally representative sample of US older adults.

## Methods

### Data sources and sample

The study used the panel data from the Core Interview and the Leave Behind Psychosocial and Lifestyle Questionnaires (LBQ) in the Health and Retirement Study (HRS) (waves during 2006–2018). In HRS, half of the samples were invited to complete the LBQ in 2006, and the other half initially started in 2008. These subsamples were followed up to complete the LBQ every four years (2006/10/14 or 2008/12/16), with follow-up rates ranging from 62 to 85%. Loneliness and social isolation were measured and generated in the LBQ. We integrated the responses in 2006 and 2008 as the first wave in this study (*n* = 13,830), and covariates were generated from core survey of the same year. In waves 2 and 3, we included participants who answered the questions about either social isolation or loneliness in the LBQ in 2010/12 (*n* = 14,791) and LBQ in 2014/16 (*n* = 13,074). For healthcare utilisation, there was no directly concurrent measure of healthcare use along with loneliness/social isolation in HRS, as core questionnaires captured individuals’ usage of services in the previous two years. Therefore, in our study, the 2-year prior/post healthcare utilisation to each LBQ measure was taken as concurrent proxy measurements of actual healthcare use. In the main analyses, we used the 2-year post healthcare indicators (from the next core questionnaire of each LBQ) as a proxy measure of concurrent healthcare use at each time point (T1-T3, with four-year intervals). Then, 2-year prior healthcare indicators (from the same core survey wave of each LBQ) were also extracted as a proxy in sensitivity analyses. Our final analytical sample was those who responded to the repeated measure of loneliness or social isolation and healthcare utilisation in three waves (*n* = 6,832). The inclusion of eligible participants in this study is illustrated in Supplementary Table 1. All participants gave informed consent.

### Measures

#### Healthcare utilisation

Healthcare utilisation included five binary measures of whether using any of the healthcare services, including inpatient care (hospital stays and re-admission to hospital), outpatient care (physician visits) and nursing home care in the past two years (yes/no). We also measured the amount of healthcare utilisation (including length of hospital stay, number of physician visits, and nights in nursing homes) in the last two years. The length of stay in hospital and nursing homes were truncated at 730 nights due to the two-year recall period [41].

#### Loneliness and social isolation

Loneliness was measured by a 3-item version of the UCLA loneliness scale, which has been validated and is comparable with the original 20-item version [42]. Participants with a score ≥ 6 were classified as being lonely [43]. We also used a continuous measure of loneliness scores in the longitudinal analysis, yielding a score range from 3 to 9 [44]. We used a 6-item social isolation index, which has been validated and applied in previous studies [13, 43, 44]. The index considered the following domains and assigned 1 point for each: (a) unmarried or not-cohabit, (b) living alone, c-e) less than monthly contact with children, with other family members, with friends, and f) nonparticipation in any groups, clubs, or other social organisations. The overall scores range from 0 to 6. As with loneliness, we applied a cut-off score of ≥ 3, as well as using the scale as a continuous measure where higher scores indicated greater levels of social isolation [44].

#### Covariates

Baseline sociodemographic information was extracted and included age, sex, race/ethnicity (White, Black, others [including American Indian, Alaskan Native, Asian or Pacific Islander, Hispanic and other]), educational attainment (None, High school, College and Postgraduate), and household wealth [quintiles]; depressive symptoms (measured using the 8-item Center for Epidemiologic Studies Depression Scale with a cut-off of ≥ 3 [yes/no]) [45, 46]; and morbidity (including stroke, diabetes, lung disease, cancer, heart conditions, high blood pressure, arthritis, or other medical condition)(yes/no).

### Statistical analysis

**Fig. 1:**
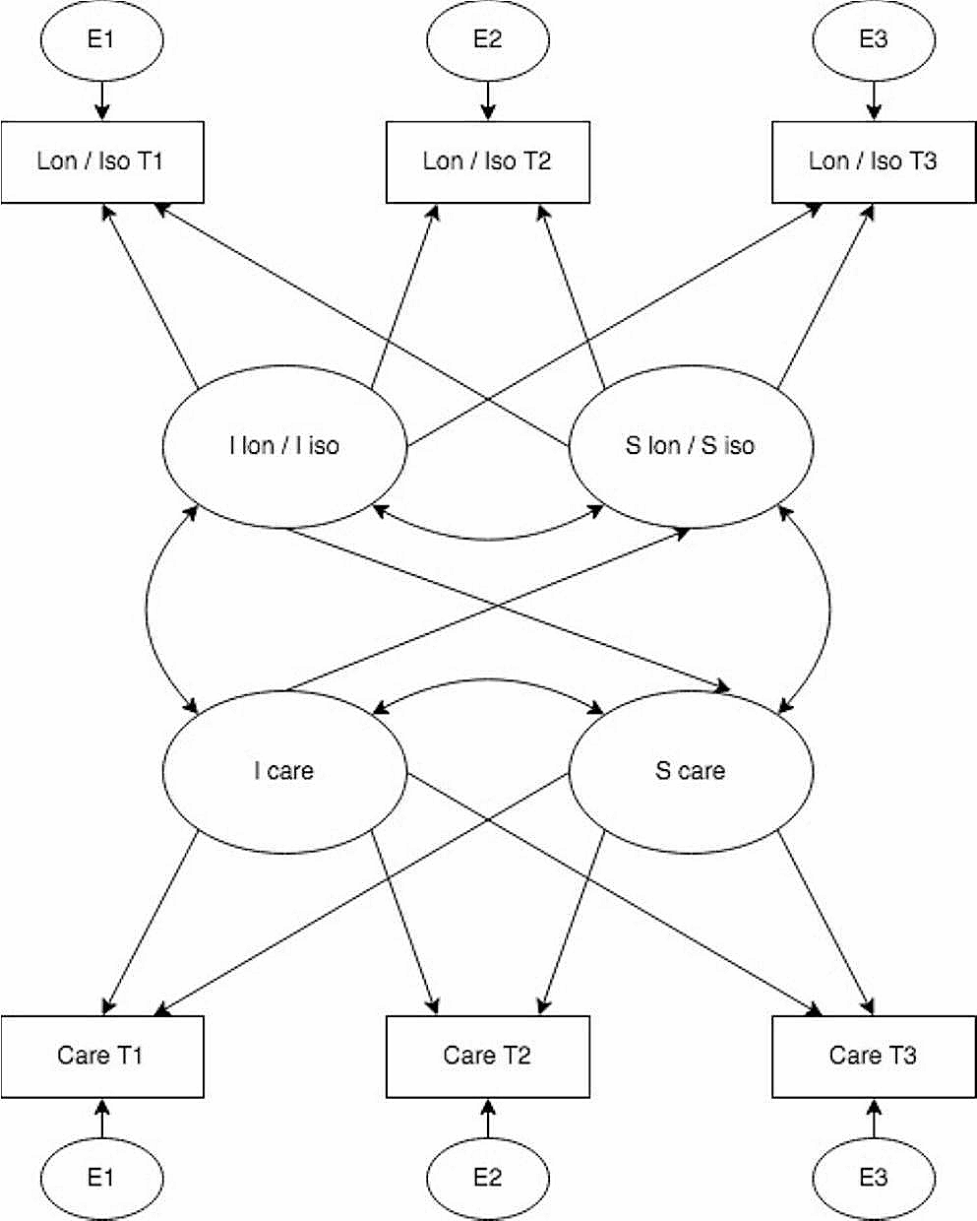
Latent growth curve models (LGCMs) for two parallel processes Note: I lon: intercept of loneliness; I iso: intercept of social isolation; I care: intercept of healthcare utilisation; S lon: slope of loneliness; S iso: slope of social isolation; S care: slope of healthcare utilisation

To estimate the relationships between trajectories of social isolation and loneliness and trajectories of healthcare utilisation over eight years, latent growth curve models (LGCMs) for two parallel processes with linear growth shapes were fitted using the continuous measures of the amount of healthcare utilisation (i.e. length of hospital stay, number of physician visits and nights in nursing homes). The linearity of growth trajectories was tested in the univariate LGCMs (Supplementary Tables 2–3). In parallel processes LGCMs, we estimated the associations between (a) baseline scores of loneliness and social isolation and healthcare utilisation at baseline; (b) baseline loneliness and social isolation and the rate of change in healthcare utilisation; (c) baseline healthcare utilisation and the rate of change in loneliness and social isolation; (d) the rate of change in loneliness and social isolation and the rate of healthcare utilisation simultaneously (Fig. 1). In estimation, age was centred at the mean, and the intercept of all studied outcomes was centred at baseline. We included crude LGCMs, partially adjusted models (adjusted for baseline age [in years], sex, ethnicity and education, and household income), and fully adjusted models (additionally adjusted for baseline depression and comorbidity). We adopted the Robust Weighted Least Squares (WLSMV) in all analyses for binary outcomes, and Maximum Likelihood Robust (MLR) estimation was applied for continuous outcomes. Model fit was assessed by the Chi-square goodness-of-fit with *p* value > 0.05 suggesting good fit [47], and the root mean square error of approximation (RMSEA) with a value of 0.06 or less indicating good fit [48]. Comparative Fit Index (CFI) [49] and Tucker-Lewis Index (TLI) [50] with values > 0.9 was considered mediocre fit and ≥ 0.95 for good fit [51]. Missingness was addressed using full information maximum likelihood (FIML) in modelling.

To further explore the relationship between baseline usage of healthcare and loneliness/social isolation trajectories, we fitted latent growth curve models (without parallel processes) using binary outcome measures as sensitivity analyses. Additionally, we conducted further sensitivity analyses testing the interaction effects of loneliness and social isolation on healthcare utilisation trajectories (including binary measures of inpatient, outpatient and nursing care usage). All analyses were conducted using the HRS LBQ weights to address non-response, making the sample representative of non-institutionalised residents in the US aged 50 years and older [52]. Analyses were performed in STATA 17.0 (StatCorp LP, Texas, USA), R Studio, version 1.4.1103 (R Project for Statistical Computing) and Mplus 8.3 (Los Angeles, CA: Muthén & Muthén).

## Results

Among weighted samples, 55.5% of the baseline participants were female, and 61.5% were aged 60 years and above. 88.0% of the sample were white, 7.6% were Black, and 4.4% were from other races/ethnic backgrounds. 21.0% of the participants had hospital stays, 6.3% were re-admitted to hospital after a discharge in the past two years, 93.9% had physician visits, and 1.2% had nursing home care (Table 1). In our sample, 24.6% were above our defined cut-off for loneliness at baseline, and 19.7% lived with social isolation. Over the eight-year follow-up, participants with social isolation or loneliness at baseline reported a higher level of inpatient and nursing home care, and lower proportions of physician visits (Supplementary Table 4).

**Table 1:** Characteristics of study populations at baseline (*n* = 6,832)

Characteristics	Overall	Social isolation			Loneliness	
		Not isolated	isolated		Not lonely	Lonely
Baseline age (Mean, SD)	63.7 (8.0)	63.4 (7.8)	64.8 (8.7)		64.1 (8.1)	62.5 (7.6)
≤ 59	38.5%	38.9%	37.0%		35.5%	47.8%
60–69	37.9%	38.8%	34.3%		39.5%	33.2%
70–79	19.1%	18.5%	21.2%		20.2%	15.5%
80 and above	4.5%	3.8%	7.6%		4.9%	3.5%
Gender						
Female	55.5%	54.8%	58.5%		54.8%	57.6%
Male	44.5%	45.2%	41.5%		45.2%	42.4%
Race/Ethnicity						
White	88.0%	87.9%	88.2%		89.5%	83.5%
Black	7.6%	7.3%	8.7%		6.7%	10.4%
Others	4.4%	4.7%	3.1%		3.9%	6.2%
Education						
None	11.6%	10.9%	14.4%		10.6%	14.7%
High school	55.2%	54.1%	59.7%		55.2%	55.5%
College	20.8%	22.0%	15.7%		21.3%	19.4%
Postgraduate	12.4%	12.9%	10.2%		13.0%	10.5%
Household income (quartiles)						
<$19,000	15.4%	11.8%	29.9%		12.6%	23.8%
$19,000-$39,999	22.0%	20.3%	29.3%		21.2%	24.6%
$40,000-$79,999	29.9%	31.4%	23.7%		30.6%	27.5%
≥$80,000	32.7%	36.5%	17.1%		35.6%	24.1%
Chronic illnesses						
No morbidity	49.2%	50.2%	45.2%		51.9%	41.0%
morbidity	50.8%	49.8%	54.8%		48.1%	59.0%
Depression						
No depression	83.3%	85.1%	76.2%		90.0%	62.8%
Depression	16.7%	14.9%	23.8%		10.0%	37.2%
Healthcare utilisation						
Hospital stays	21.0%	20.6%	22.9%		20.3%	23.4%
Length of hospital stay (Mean, SD)	1.2 (4.5)	1.1 (3.8)	1.7 (6.8)		1.1 (3.8)	1.5 (6.1)
Readmission to hospital	6.3%	6.0%	7.9%		6.0%	7.5%
Physician visits	93.9%	94.5%	91.4%		94.6%	91.7%
Times of physician visits (Mean, SD)	8.9 (12.9)	8.8 (12.5)	9.6 (14.6)		8.4 (11.6)	10.5 (16.1)
Nursing home care	1.2%	0.8%	2.6%		1.1%	1.4%
Nights in nursing home (Mean, SD)	0.5 (9.7)	0.2 (3.5)	1.6 (20.6)		0.3 (5.1)	0.9 (16.9)

### Main analyses

Baseline loneliness was correlated with higher levels of all types of baseline healthcare. However, after adjusting for baseline depression and morbidity, only the relationship with baseline outpatient care remained (physician visits *β* = 0.06, *SE* = 0.03). In contrast, baseline social isolation was associated with higher levels of just inpatient care and nursing home care, with these results maintaining even in fully adjusted models (length of hospital stay *β* = 0.07, *SE* = 0.04; length of nursing home stay *β* = 0.05, *SE* = 0.02).

There was no association between trajectories of loneliness or isolation and trajectories of healthcare utilisation. Nor did baseline loneliness or isolation levels predict trajectories of healthcare utilisation. However, the intercept of nights in nursing homes predicted the rate of change in loneliness (*β*= -0.15, *SE* = 0.07) and social isolation (*β*= -0.04, *SE* = 0.02) over the 8 years, indicating better trajectories of loneliness and isolation for people who spent longer in nursing homes (Table 2).

Model fit for all the adjusted models performed well, ranging from models for inpatient and outpatient care (CFI = 1.0, TLI = 1.0, RMSEA 0.01 (90%CI 0.00-0.01) to (CFI = 0.98, TLI = 0.94, RMSEA 0.02 (90%CI 0.02–0.03) for loneliness and nursing home care model (Supplementary Table 5).

### Sensitivity analyses

We explored further the relationship between baseline healthcare utilisation and trajectories of loneliness and isolation using binary healthcare measures. Baseline loneliness was related to a lower likelihood that older adults visited a physician at all (*β*= -0.15, *SE* = 0.08) but not other types of healthcare, while baseline social isolation was related to a lower likelihood of whether older adults made any use of outpatient care (*β*= -0.19, *SE* = 0.08), but a higher likelihood of them using nursing home care (*β* = 0.40, *SE* = 0.12). The relationship between baseline nursing home care and the trajectory of social isolation was also maintained (*β*= -0.39, *SE* = 0.19). All LGCMs had a good model fit (Supplementary Table 6). Finally, when interacting social isolation and loneliness, no relationship was found with baseline levels or trajectories of any healthcare utilisation (Supplementary Table 7). The results for the proxy measurement using 2-year prior healthcare utilisation showed comparable effect sizes and similar trends of the associations using 2-year post proxy measurement of healthcare, but with most relationships attenuated after adjusting for confounders (Supplementary Tables 8–11).

**Table 2:** Longitudinal associations between loneliness, social isolation, and healthcare utilisation trajectories over eight years

Loneliness	Inpatient care (Length of hospital stay)	Outpatient care (Numbers of physical visits)	Nursing home care (Nights in nursing home)	Social isolation		Inpatient care (Length of hospital stay)	Outpatient care (Numbers of physical visits)	Nursing home care (Nights in nursing home)
Unadjusted model	*β (SE)*	*β (SE)*	*β (SE)*	Unadjusted model		*β (SE)*	*β (SE)*	*β (SE)*
I lon & I care	0.12 (0.04) **	0.13 (0.03) ***	0.08 (0.03) *	I iso & I care	0.14 (0.04) ***		0.03 (0.02)	0.07 (0.02) **
S lon & S care	-0.01(0.22)	0.05 (0.06)	0.25 (0.21)	S iso & S care	0.06 (0.12)		-0.02 (0.03)	0.22 (0.14)
I lon →S care	0.15 (0.12)	-0.04 (0.04)	-0.13 (0.08)	I iso → S care	0.12 (0.09)		-0.04 (0.04)	0.04 (0.07)
I care → S lon	0.02 (0.09)	0.01(0.06)	-0.17 (0.08) *	I care → S iso	-0.02 (0.05)		-0.002 (0.03)	-0.03 (0.02)
Partially adjusted model				Partially adjusted model				
I lon & I care	0.11 (0.05) *	0.14 (0.03) ***	0.08 (0.03) *	I iso & I care		0.08 (0.04) *	0.01 (0.02)	0.05 (0.02) **
S lon & S care	-0.02 (0.23)	0.05 (0.06)	0.15 (0.15)	S iso & S care		0.02 (0.11)	-0.02 (0.03)	0.13 (0.09)
I lon → S care	0.17 (0.12)	-0.04 (0.04)	-0.08 (0.07)	I iso → S care		0.11 (0.10)	-0.03 (0.04)	-0.001 (0.05)
I care → S lon	0.03 (0.10)	-0.01 (0.06)	-0.17 (0.08) *	I care → S iso		-0.04 (0.05)	-0.03 (0.03)	-0.04 (0.02) *
Fully adjusted model				Fully adjusted model				
I lon & I care	0.05 (0.04)	0.06 (0.03) *	0.06 (0.03)	I iso & I care		0.07 (0.04) *	-0.01(0.02)	0.05 (0.02) **
S lon & S care	-0.02 (0.21)	0.05 (0.05)	0.14 (0.14)	S iso & S care		0.01 (0.11)	-0.02 (0.03)	0.13 (0.09)
I lon → S care	0.14 (0.11)	0.01 (0.05)	-0.08 (0.09)	I iso → S care		0.11 (0.09)	-0.02 (0.04)	-0.001(0.05)
I care → S lon	0.04 (0.10)	0.01 (0.06)	-0.15 (0.07) *	I care → S iso		-0.04 (0.05)	-0.04 (0.03)	-0.04 (0.02) *

Note. *p < 0.05, **p < 0.01, ***p < 0.001. β, Standardised estimates. The estimates were based on loneliness and social isolation scores. I lon, intercept of loneliness; I iso, intercept of social isolation; I care, intercept of healthcare utilisation; S lon, slope of loneliness; S iso, slope of social isolation; S care, slope of healthcare utilisation. Double-headed arrows refer to correlations. Single-headed arrows represent regression effects. Partially adjusted models adjusted for baseline age, gender, ethnicity, education and household income. Fully adjusted models additionally adjusted for baseline depression and morbidity

## Discussion

This study explored the longitudinal relationships between social isolation, loneliness, and healthcare utilisation trajectories. Confirming some previous work, both loneliness and social isolation were related to multiple types of healthcare utilisation cross-sectionally. But our results showed three key new findings, all of which highlight the complexity of the relationship between social deficits and healthcare. First, the cross-sectional relationship between loneliness and inpatient care (whether in a hospital or care home) was explained through physical and mental health in older adults, with only the relationship with outpatient care remaining. Second, after adjusting for socio-demographics and health, loneliness and social isolation were both cross-sectionally related to a lower binary likelihood of seeking outpatient care. However, loneliness was also related to a higher usage of outpatient care (suggested a non-linear relationship), while isolation was related to a higher usage of inpatient care (both length of hospital admission and nursing home care). Third, when exploring the direction of this relationship, our results suggest that nursing home stays may be a stronger trigger for subsequent trajectories of loneliness and isolation, rather than the reverse. But there was no clear evidence that social deficits led to specific trajectories of healthcare utilisation, nor vice versa.

The finding that the relationship between loneliness and both inpatient and nursing home care was attenuated when accounting for mental and physical health suggests that the relationship is primarily derived from diseases and health status. This echoes previous evidence that loneliness may have limited impacts on general or planned inpatient admissions, as health status may mediate the associations between social deficits (loneliness and social isolation) and hospitalisation [32, 34, 36]. Older people living with chronic health conditions are at a greater risk of experiencing loneliness, and in turn, loneliness could damage their health status [10, 20, 21, 53]. Biological mediators of this relationship have also previously been demonstrated via poorer regulation of inflammation [54]. The potential loop may accumulate adverse health effects in the long term, so loneliness may act as a more chronic risk factor for specialised and institutional-based healthcare (i.e. inpatient and nursing home care) [36].

In contrast, our findings that social isolation but not loneliness was associated with more frequent nursing home admissions and prolonged institutional health stays independent of health confounders support some previous findings [34]. Social isolation may have an intensive role in triggering extended inpatient and nursing home stays due to the adverse health effects of social isolation [43] and worsening disease management [3, 19]. Interestingly, we found that when using a binary measure of healthcare utilisation, socially isolated older adults were less likely to seek outpatient support, suggesting that they may be delaying or ignoring symptoms, meaning that opportunities for prevention are missed, potentially accumulating institutional care demands [26]. Similarly, consistent with previous evidence, we found an association between social isolation and extended hospital stay, but not admissions to hospital [32, 34, 36, 40]. Exceptions in previous literature focus on more frequent hospital admission for chronic and respiratory diseases [37, 38]. Hospital admission is primarily triggered by illness severity rather than patient decision, while length of hospital stay can be affected by factors attributable to social isolation such as recovery rate (e.g. a lack of positive coping strategies) and challenges in where to discharge the patient to (e.g. lack of suitable housing or social support at home) [9, 20, 21, 25]. Socially isolated older people may have to alleviate unmet social needs through nursing home care usage [27].

Aligning with previous longitudinal findings [30, 39], loneliness and social isolation were both associated with fewer physician visits regardless of sociodemographic and health status, suggesting the obstacle of social deficits in accessing outpatient care. Older adults with social deficits are more likely to hold negative stereotypes of healthy ageing, including ageism [22], poorer self-rated health status [23] and lower expectations of longevity [24], potentially preventing the development of healthy healthcare-seeking behaviours. For the number of physician visits, only loneliness but not social isolation was associated with increased numbers of physician visits in our study, complementing previous cross-sectional and longitudinal evidence [33–36]. Lonely older individuals are at higher risks of living with multiple chronic conditions, such as cognitive declines and dementia [55, 56], intrinsic capacity [3, 4], somatic symptoms [57], chronic pain [58] and poorer mental well-being [20], which could aggregate health needs. However, unmet psychological needs (e.g. subjective or non-clinical) may also drive a need for more frequent social interaction with health providers [26, 27], leading to potential overutilisation of healthcare.

Notably, our findings that baseline nursing home stays are associated with higher loneliness and isolation confirm and extend previous research [59–61]. The geographical and social separation of older people and their familiar social network (e.g. family, friends and neighbours) and lack of communication and belonging after nursing home enrolment and formation of more superficial rather than deep or emotional relationships could increase social deficits. However, the current findings also suggest that extended nursing home stays predicted better trajectories of loneliness and isolation over time, which could be partially explained through nursing homes providing more daily contact than for older adults living alone in the community. Our finding that longer nursing home stays also related to better loneliness trajectories goes against some previous studies suggesting that nursing homes may provide social contact but not meet emotional needs, but our findings on this factor were less robust to sensitivity analyses [27, 62, 63].

A main strength of this study lies in using a longitudinal and nationally representative cohort of older Americans, which involves validated measures of loneliness, social isolation, and a wide range of healthcare services. Beyond examining the relationships between inpatient, outpatient, nursing home admission and loneliness and social isolation trajectories, we fitted LGCMs for two parallel pathways, which allows for estimating the trajectories of loneliness and social isolation and the amount of healthcare usage simultaneously. The modelling advantage the understanding of changes in both factors over eight years. However, the current estimates may be limited by the available waves of LBQ in the HRS (i.e. three waves), and the nature of LGCMs cannot warrant causality. Besides, the relatively higher follow-up attrition of older participants and those living with lower household income and health conditions may introduce selection bias and reverse causality (Supplementary Table 12). However, we applied the FIML estimation based on weighted data, which can minimise selective bias. Another limitation was that the current estimates only adjusted for time-invariant covariates. The unobserved bias in our models remains, and we cannot rule out the possibility of uncontrolled confounders in our models. Health-related factors may mediate the examined relationships, leading to over-adjustment bias. Further prospective and experimental investigations are needed to explore the courses of loneliness and social isolation and interplays with healthcare utilisation over time, especially using more validated and frequent monitoring (e.g., health records). Qualitative evidence exploring the underlying mechanisms of these links could also provide key policy implications. Finally, a key challenge in measuring social deficits and healthcare utilisation relates to the timing of measures. Social deficits were asked for present circumstances whereas healthcare utilisation explored timepoints over two years. The direction of our findings was the same whether focusing on past recollections of healthcare utilisation and examining patterns for the forthcoming time period, but the statistical significance of findings varied. Given the complexity of the longitudinal patterns found in these analyses, future studies are encouraged that test the replicability of the findings presented here and the degree to which recall bias affects the stability of the results.

## Conclusion

The longitudinal findings shed light on the associations of loneliness, social isolation, and healthcare utilisation trajectories over eight years. Our results show a complex bidirectional relationship between loneliness, social isolation and health needs. Both types of social deficits may be initial barriers to accessing preventative outpatient healthcare, with subjective feelings of isolation also related to more frequent physician visits, and objective measures of isolation related to extended inpatient and nursing home care independent of sociodemographic and health status. Social isolation and loneliness could therefore exacerbate the negative loop of poor health conditions and increased healthcare use, further intensifying the unmet health needs at the populational level and burdening the equity of health resource allocations. Thus, delivering complex non-clinical interventions could be a holistic way to break the cycle. However, nursing home care may support future trajectories, especially of social isolation. Overall, the findings echo previous evidence that societal non-clinical services (i.e. social prescribing) have the potential to address health and social needs holistically and reduce health demands for secondary care services [64]. Embedding non-clinical services into the healthcare system can partially address healthcare needs driven by social deficits and facilitate protective health-seeking behaviours. The study supports the necessity for enlarging policy inputs to develop and integrate social prescribing in the health system, allowing better achieving the goal of healthy ageing.

## Electronic supplementary material

Below is the link to the electronic supplementary material.

Supplementary Material 1
