# Supplementary material for: Longitudinal associations between loneliness, social isolation, and healthcare utilisation trajectories: a latent growth curve analysis
Source: Soc Psychiatry Psychiatr Epidemiol. 2024 Mar 1;59(10):1839–48. doi: 10.1007/s00127-024-02639-9 (PMC11464645; doi:10.1007/s00127-024-02639-9)
Supplement: Supplementary file 1 — Supplementary Material 1 [file 127_2024_2639_MOESM1_ESM.docx]

**Supplementary materials for: Longitudinal associations between loneliness, social isolation, and healthcare utilisation trajectories: a latent growth curve analysis**

Social Psychiatry and Psychiatric Epidemiology

Qian Gao, PhD ^1^, Hei Wan Mak, PhD ^1^ and Daisy Fancourt, PhD ^1*^

1. Research Department of Behavioural Science and Health, Institute of Epidemiology & Health Care, University College London, London, UK

***Corresponding author:** Daisy Fancourt. Email: [d.fancourt@ucl.ac.uk](mailto:d.fancourt@ucl.ac.uk)

**Supplementary Table 1.** The study sample selection process

**Supplementary Table 2.** Univariate latent growth curve models (LGCMs)

**Supplementary Table 3.** Sample and estimated means for each outcome measure

**Supplementary Table 4.** The prevalence of different types of healthcare utilisation over an 8-year follow-up by loneliness and social isolation (n=6,832)

**Supplementary Table 5.** Model fit for unadjusted, partially, and fully adjusted LGCMs models

**Supplementary Figure 1.** The individual scores of social isolation and loneliness over three waves

**Sensitivity analyses:**

**Supplementary Table 6.** Associations between loneliness and social isolation trajectories and baseline healthcare utilisation

**Supplementary Table 7.** Longitudinal associations between baseline loneliness and social isolation (including interaction term) and healthcare utilisation trajectories

**Supplementary Table 8.** Longitudinal associations between loneliness, social isolation, and healthcare utilisation (2-year prior healthcare) trajectories over eight years

**Supplementary Table 9.** Model fit for unadjusted, partially, and fully adjusted LGCMs models

**Supplementary Table 10.** Associations between loneliness and social isolation trajectories and baseline healthcare utilisation (2-year prior healthcare)

**Supplementary Table 11.** Longitudinal associations between baseline loneliness and social isolation (including interaction term) and healthcare utilisation (2-year prior healthcare) trajectories

**Supplementary Table 12.** Baseline characteristics of samples at baseline and longitudinal sample

**Supplementary Table 1.** The study sample selection process

| *Main analyses* |  | |  | |  | |
| --- | --- | --- | --- | --- | --- | --- |
| **Waves** | **Wave 1 (T1)** | | **Wave 2 (T2)** | | **Wave 3 (T3)** | |
| Year | 2006 | 2008 | 2010 | 2012 | 2014 | 2016 |
| Eligible for LBQ | 8,681 | 8,296 | 11,213 | 10,079 | 9,549 | 10,238 |
| Completed LBQ | 7,236 | 6,594 | 7,828 | 6,963 | 6,973 | 6,101 |
| Responded to loneliness/social isolation | 13,830 | | 14,791 | | 13,074 | |
| Healthcare utilisation captured from the following core survey of each LBQ | Core survey (2008/10)  12,593 | | Core survey (2012/14)  13,591 | | Core survey (2016/18)  11,400 | |
| Responded to all three waves | Final sample (n=6,832) | | | | | |
| *Sensitivity analyses* |  | | | | | |
| **Waves** | **Wave 1 (T1)** | | **Wave 2 (T2)** | | **Wave 3 (T3)** | |
| Year | 2006 | 2008 | 2010 | 2012 | 2014 | 2016 |
| Eligible for LBQ | 8,681 | 8,296 | 11,213 | 10,079 | 9,549 | 10,238 |
| Completed LBQ | 7,236 | 6,594 | 7,828 | 6,963 | 6,973 | 6,101 |
| Responded to loneliness/social isolation | 13,830 | | 14,791 | | 13,074 | |
| Healthcare utilisation captured from the following core survey of each LBQ | Core survey (2006/8)  13,829 | | Core survey (2010/12)  14,772 | | Core survey (2014/16)  13,062 | |
| Responded to all three waves | Final sample (n=6,832) | | | | | |

**Supplementary Table 2.** Univariate latent growth curve models (LGCMs)

| *Main analyses* |  |  |  |  |  |  |
| --- | --- | --- | --- | --- | --- | --- |
| Outcomes | Mean intercept | Mean slope | Variance intercept | Variance slope | Correlation between intercept and slope (r) | (SE) |
| Loneliness | 4.34 *** | -0.01 *** | 1.64 *** | 0.01 *** | -0.35 *** | 0.05 |
| Social isolation | 1.55 *** | 0.03 *** | 0.81 *** | 0.01 *** | -0.28 *** | 0.03 |
| Length of hospital stay (2-year post) | 1.17 *** | 0.09 *** | 3.80*** | 0.08 | -0.42 * | 0.21 |
| Times of physician visits (2-year post) | 9.06 *** | 0.10 ** | 87.94 *** | 1.28 *** | -0.52 *** | 0.09 |
| Nights in nursing home (2-year post) | 0.42 *** | 0.25 *** | 39.25 | 0.71 | -0.84 *** | 0.19 |
| *Sensitivity analyses* |  |  |  |  |  |  |
| Loneliness | 4.34 *** | -0.01 *** | 1.64 *** | 0.01 *** | -0.35 *** | 0.05 |
| Social isolation | 1.55 *** | 0.03 *** | 0.81 *** | 0.01 *** | -0.28 *** | 0.03 |
| Length of hospital stay (2-year prior) | 1.17 *** | 0.09 *** | 1.71 | 0.01 | 1.18 | 0.31 |
| Times of physician visits (2-year prior) | 9.25 *** | 0.17 *** | 102.22 *** | 1.33 * | -0.46 | 0.27 |
| Nights in nursing home (2-year prior) | 0.20 * | 0.08 *** | 1.81 | 0.13 * | -0.04 *** | 0.14 |

*Note*. *p < 0.05, **p < 0.01, ***p < 0.001, the value of the slope reflects the yearly change in the outcome measure. Results presented are based on univariate LGCMs for each outcome measure, after adjustment for baseline age, gender, ethnicity, education, household income, depression and morbidity.

**Supplementary Table 3.** Sample and estimated means for each outcome measure

| *Main analyses* | **wave1** | **wave2** | **wave3** | *Sensitivity analyses* | **wave1** | **wave2** | **wave3** |
| --- | --- | --- | --- | --- | --- | --- | --- |
| **Sample means** |  |  |  | **Sample means** |  |  |  |
| Loneliness | 4.35 | 4.26 | 4.25 | Loneliness | 4.35 | 4.26 | 4.25 |
| Social isolation | 1.55 | 1.69 | 1.79 | Social isolation | 1.55 | 1.69 | 1.79 |
| Length of hospital stay | 1.19 | 1.48 | 2.04 | Length of hospital stay | 1.17 | 1.52 | 1.94 |
| Times of physician visits | 9.06 | 9.46 | 9.85 | Times of physician visits | 9.13 | 10.43 | 10.40 |
| Nights in nursing home | 0.46 | 1.03 | 3.94 | Nights in nursing home | 0.25 | 0.43 | 0.95 |
| **Model estimated means** |  |  |  | **Model estimated means** |  |  |  |
| Loneliness | 4.34 | 4.29 | 4.25 | Loneliness | 4.34 | 4.29 | 4.25 |
| Social isolation | 1.55 | 1.68 | 1.80 | Social isolation | 1.55 | 1.68 | 1.80 |
| Length of hospital stay | 1.17 | 1.54 | 1.91 | Length of hospital stay | 1.17 | 1.54 | 1.91 |
| Times of physician visits | 9.06 | 9.45 | 9.84 | Times of physician visits | 9.25 | 9.94 | 10.63 |
| Nights in nursing home | 0.42 | 1.42 | 2.41 | Nights in nursing home | 0.20 | 0.52 | 0.84 |

*Note.* Fully adjusted Models

**Supplementary Table 4.** The prevalence of different types of healthcare utilisation over an 8-year follow-up by loneliness and social isolation (n=6,832)

| **Loneliness** | **Wave1** |  | **Wave2** |  | **Wave3** |  |
| --- | --- | --- | --- | --- | --- | --- |
| **Inpatient care** | Not lonely | lonely | Not lonely | lonely | Not lonely | lonely |
| Hospital stays | 20.3% | 23.4% | 22.1% | 25.2% | 24.2% | 29.1% |
| Readmission to hospital (2-year) | 6.0% | 7.5% | 6.6% | 9.5% | 9.2% | 11.7% |
| **Outpatient care**- physician visits | 94.6% | 91.7% | 93.3% | 93.3% | 94.1% | 92.2% |
| **Nursing home care** | 1.1% | 1.4% | 1.9% | 2.8% | 4.1% | 5.4% |
| **Overall** | 75.4% | 24.6% | 77.3% | 22.7% | 77.4% | 22.6% |
| **Social isolation** |  |  |  |  |  |  |
| **Inpatient care** | Not isolated | Isolated | Not isolated | Isolated | Not isolated | Isolated |
| Hospital stays | 20.6% | 22.9% | 21.6% | 26.5% | 24.2% | 28.3% |
| Readmission to hospital (2-year) | 6.0% | 7.9% | 6.5% | 9.5% | 8.9% | 12.2% |
| **Outpatient care**- physician visits | 94.5% | 91.4% | 93.6% | 92.4% | 94.0% | 92.7% |
| **Nursing home care** | 0.8% | 2.6% | 1.5% | 4.0% | 3.2% | 7.4% |
| **Overall** | 80.3% | 19.7% | 75.6% | 24.4% | 71.5% | 28.5% |

**Supplementary Table 5.** Model fit for unadjusted, partially, and fully adjusted LGCMs models

|  | **Loneliness** | | | | **Social isolation** | | | |
| --- | --- | --- | --- | --- | --- | --- | --- | --- |
| **Length of hospital stay** | chi^2^(df) | CFI | TLI | RMSEA (90% CI) | chi^2^(df) | CFI | TLI | RMSEA (90% CI) |
| Unadjusted model | 9.85 (7) | 1.0 | 1.0 | 0.01 (0.00-0.02) | 12.39 (7) | 1.0 | 1.0 | 0.01 (0.00-0.02) |
| Partially adjusted model | 20.73 (17) | 1.0 | 1.0 | 0.01 (0.00-0.01) | 26.21 (17) | 1.0 | 1.0 | 0.01 (0.00-0.02) |
| Fully adjusted model | 27.34 (21) | 1.0 | 1.0 | 0.01 (0.00-0.01) | 27.53 (21) | 1.0 | 1.0 | 0.01 (0.00-0.01) |
| **Times of physician visits** |  |  |  |  |  |  |  |  |
| Unadjusted model | 8.83 (7) | 1.0 | 1.0 | 0.01 (0.00-0.02) | 9.45 (7) | 1.0 | 1.0 | 0.01 (0.00-0.02) |
| Partially adjusted model | 21.05 (17) | 1.0 | 1.0 | 0.01 (0.00-0.01) | 22.76 (17) | 1.0 | 1.0 | 0.01 (0.00-0.01) |
| Fully adjusted model | 30.05 (21) | 1.0 | 1.0 | 0.01 (0.00-0.01) | 26.31 (21) | 1.0 | 1.0 | 0.01 (0.00-0.01) |
| **Nights in nursing home** |  |  |  |  |  |  |  |  |
| Unadjusted model | 18.38 (7) | 0.99 | 0.98 | 0.02 (0.01-0.02) | 32.46 (7) | 0.99 | 0.97 | 0.02 (0.02-0.03) |
| Partially adjusted model | 85.66 (17) | 0.97 | 0.93 | 0.02 (0.02-0.03) | 115.70 (17) | 0.98 | 0.94 | 0.03 (0.02-0.03) |
| Fully adjusted model | 91.90 (21) | 0.98 | 0.94 | 0.02 (0.02-0.03) | 110.18 (21) | 0.98 | 0.95 | 0.03 (0.02-0.03) |

*Note.* Model fit for Table 2. Partially adjusted models adjusted for baseline age, gender, ethnicity, education and household income. Fully adjusted models additionally adjusted for baseline depression and morbidity.

**Supplementary Figure 1.** The individual scores of social isolation and loneliness over three waves

**
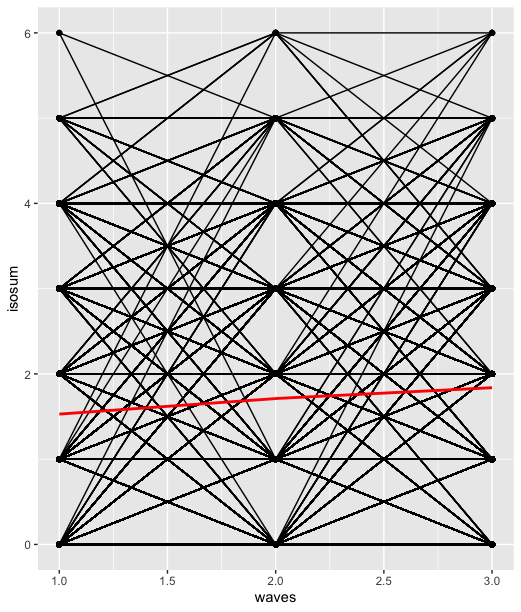

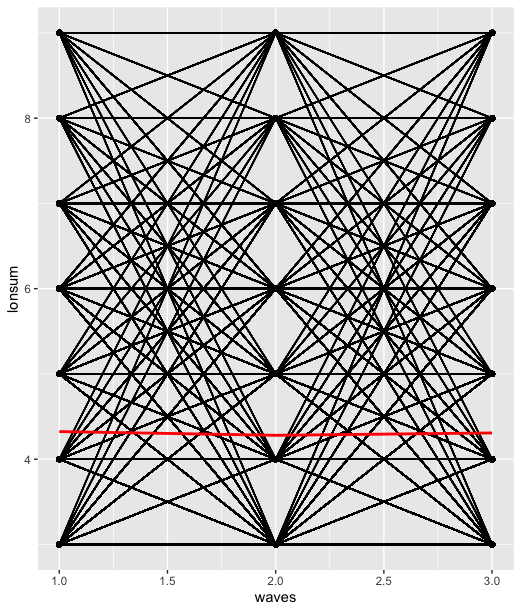
**

(a) Social isolation scores over three waves (b) Loneliness scores over three waves

**Sensitivity analyses:**

**Supplementary Table 6.** Associations between loneliness and social isolation trajectories and baseline healthcare utilisation

|  | **Inpatient care** |  | **Outpatient care** | **Nursing home care** |
| --- | --- | --- | --- | --- |
|  | hospitalisation | re-hospitalisation | physician visits |  |
| **Loneliness trajectory** | *β (SE)* | *β (SE)* | *β (SE)* | *β (SE)* |
| *I lon* | 0.01 (0.04) | 0.03 (0.06) | -0.15 (0.08) * | 0.11 (0.14) |
| *S lon* | 0.07 (0.08) | 0.08 (0.14) | 0.12 (0.16) | 0.05 (0.31) |
| Model fit |  |  |  |  |
| chi2(df) | 19.70 (9) | 20.35 (9) | 19.69 (9) | 20.21 (9) |
| CFI | 1.0 | 1.0 | 1.0 | 1.0 |
| TLI | 1.0 | 1.0 | 1.0 | 1.0 |
| RMSEA (90% CI) | 0.01 (0.01-0.02) | 0.01 (0.01-0.02) | 0.01 (0.01-0.02) | 0.01 (0.01-0.02) |
| **Social isolation trajectory** | *β (SE)* | *β (SE)* | *β (SE)* | *β (SE)* |
| *I iso* | -0.01 (0.04) | 0.10 (0.06) | -0.19 (0.08) * | 0.40 (0.12) ** |
| *S iso* | -0.04 (0.05) | -0.15 (0.08) | -0.07 (0.11) | -0.39 (0.19) * |
| Model fit |  |  |  |  |
| chi2(df) | 13.53 (9) | 13.84 (9) | 13.15 (9) | 14.75 (9) |
| CFI | 1.0 | 1.0 | 1.0 | 1.0 |
| TLI | 1.0 | 1.0 | 1.0 | 1.0 |
| RMSEA (90% CI) | 0.01 (0.00-0.02) | 0.01 (0.00-0.02) | 0.01 (0.00-0.02) | 0.01 (0.00-0.02) |

*Note.* *p < 0.05, **p < 0.01, ***p < 0.001. β, Standardised estimates. We applied a cut-off of scores ≥3 for loneliness and a cut-off of ≥6 for social isolation in the estimates. I lon, intercept of loneliness; I care, intercept of healthcare utilisation; S lon, slope of loneliness; S care, slope of healthcare utilisation. Single-headed arrows represent regression effects. All models adjusted for baseline age, gender, ethnicity, education, household income, depression and morbidity.

**Supplementary Table 7.** Longitudinal associations between baseline loneliness and social isolation (including interaction term) and healthcare utilisation trajectories

|  | **Inpatient care** |  | **Outpatient care** | **Nursing home care** |
| --- | --- | --- | --- | --- |
|  | hospital stays | re-admission to hospital | physician visits | nursing home stays |
| **loneliness & social isolation** | *β (SE)* | *β (SE)* | *β (SE)* | *β (SE)* |
| loneliness I care | 0.13 (0.11) | 0.07 (0.13) | 0.06 (0.13) | NC |
| loneliness S care | -0.10 (0.16) | 0.07 (0.21) | NC | NC |
| social isolation I care | -0.09 (0.16) | -0.26 (0.19) | -0.29 (0.18) | NC |
| social isolation S care | 0.13 (0.25) | 0.33 (0.24) | NC | NC |
| Iso* lon I care | 0.02 (0.33) | -0.24 (0.38) | 0.38 (0.47) | NC |
| Iso* lon S care | 0.20 (0.65) | 0.23 (0.51) | NC | NC |
| Model fit |  |  |  |  |
| chi2(df) | 7.61 (9) | 5.98 (9) | 3.89 (9) | NC |
| CFI | 1.0 | 1.0 | 1.0 | NC |
| TLI | 1.0 | 1.0 | 1.0 | NC |
| RMSEA (90% CI) | <0.01 (0.00-0.01) | <0.01 (0.00-0.01) | <0.01 (0.00-0.01) | NC |

*Note.* *p < 0.05, **p < 0.01, ***p < 0.001. β, Standardised estimates. We applied a cut-off of scores ≥3 for loneliness and a cut-off of ≥6 for social isolation in the estimates. I lon, intercept of loneliness; I care, intercept of healthcare utilisation; S lon, slope of loneliness; S care, slope of healthcare utilisation. Single-headed arrows represent regression effects; Models adjusted for baseline age, gender, ethnicity, education, household income, and additionally adjusted for baseline depression and morbidity.

**Supplementary Table 8.** Longitudinal associations between loneliness, social isolation, and healthcare utilisation (2-year prior healthcare) trajectories over eight years

| **Loneliness** | | **Inpatient care**  (Length of hospital stay) | **Outpatient care**  (Numbers of physical visits) | | **Nursing home care**  (Nights in nursing home) | | **Social isolation** | | **Inpatient care**  (Length of hospital stay) | **Outpatient care**  (Numbers of physical visits) | | **Nursing home care**  (Nights in nursing home) | |
| --- | --- | --- | --- | --- | --- | --- | --- | --- | --- | --- | --- | --- | --- |
| Unadjusted model | | *β (SE)* | *β (SE)* | | *β (SE)* | | Unadjusted model | | *β (SE)* | *β (SE)* | | *β (SE)* | |
| I lon & I care | 0.28 (0.12) * | | | 0.13 (0.03) *** | | 0.05 (0.06) | I iso & I care | 0.19 (0.11) | | | 0.01 (0.02) | | 0.10 (0.10) |
| S lon & S care | 0.67(2.59) | | | 0.02 (0.08) | | 0.08 (0.06) | S iso & S care | -0.51 (2.53) | | | -0.002 (0.04) | | 0.06 (0.04) |
| I lon S care | -0.04 (0.29) | | | -0.02 (0.05) | | -0.02 (0.03) | I iso S care | 0.003 (0.26) | | | -0.03 (0.04) | | 0.05 (0.04) |
| I care S lon | -0.14 (0.19) | | | -0.09 (0.07) | | -0.02 (0.10) | I care S iso | 0.13 (0.15) | | | 0.03 (0.03) | | 0.01 (0.04) |
| Partially adjusted model | | | | | | | Partially adjusted model | | | | | | |
| I lon & I care | | 0.26 (0.13) * | 0.13 (0.03) *** | | 0.07 (0.09) | | I iso & I care | | 0.07 (0.08) | -0.02 (0.02) | | 0.07 (0.11) | |
| S lon & S care | | 0.64 (2.36) | 0.02 (0.09) | | 0.04 (0.07) | | S iso & S care | | -0.56 (2.61) | -0.004 (0.04) | | 0.01 (0.04) | |
| I lon S care | | 0.001 (0.20) | -0.01 (0.05) | | 0.02 (0.04) | | I iso S care | | -0.03 (0.26) | -0.02 (0.04) | | 0.05 (0.05) | |
| I care S lon | | -0.13 (0.21) | -0.10 (0.07) | | -0.04 (0.12) | | I care S iso | | 0.14 (0.17) | 0.01 (0.03) | | -0.02 (0.05) | |
| Fully adjusted model | | | | | | | Fully adjusted model | | | | | | |
| I lon & I care | | 0.15 (0.12) | 0.04 (0.03) | | 0.05 (0.07) | | I iso & I care | | 0.05 (0.09) | -0.04 (0.03) | | 0.06 (0.11) | |
| S lon & S care | | 0.48 (1.54) | 0.01 (0.08) | | 0.03 (0.07) | | S iso & S care | | -0.52 (1.91) | -0.003 (0.04) | | 0.01 (0.04) | |
| I lon S care | | 0.10 (0.24) | 0.03 (0.05) | | 0.01 (0.05) | | I iso S care | | 0.02 (0.19) | -0.01 (0.04) | | 0.05 (0.05) | |
| I care S lon | | -0.13 (0.26) | -0.08 (0.06) | | -0.03 (0.12) | | I care S iso | | 0.19 (0.25) | 0.01 (0.03) | | -0.02 (0.05) | |

*Note.* *p < 0.05, **p < 0.01, ***p < 0.001. β, Standardised estimates. The estimates were based on Loneliness and social isolation scores. I lon, intercept of loneliness; I iso, intercept of social isolation; I care, intercept of healthcare utilisation; S lon, slope of loneliness; S iso, slope of social isolation; S care, slope of healthcare utilisation. Double-headed arrows refer to correlations. Single-headed arrows represent regression effects. Partially adjusted models adjusted for baseline age, gender, ethnicity, education and household income. Fully adjusted models additionally adjusted for baseline depression and morbidity.

**Supplementary Table 9.** Model fit for unadjusted, partially, and fully adjusted LGCMs models

|  | **Loneliness** | | | | **Social isolation** | | | |
| --- | --- | --- | --- | --- | --- | --- | --- | --- |
| **Length of hospital stay** | chi^2^(df) | CFI | TLI | RMSEA (90% CI) | chi^2^(df) | CFI | TLI | RMSEA (90% CI) |
| Unadjusted model | 7.65 (7) | 1.0 | 1.0 | 0.00 (0.00-0.02) | 9.45 (7) | 1.0 | 1.0 | 0.01 (0.00-0.02) |
| Partially adjusted model | 24.10 (17) | 1.0 | 0.99 | 0.01 (0.00-0.01) | 29.16 (17) | 1.0 | 0.99 | 0.01 (0.00-0.02) |
| Fully adjusted model | 31.75 (21) | 1.0 | 0.99 | 0.01 (0.00-0.02) | 31.11 (21) | 1.0 | 0.99 | 0.01 (0.00-0.01) |
| **Times of physician visits** |  |  |  |  |  |  |  |  |
| Unadjusted model | 22.38 (7) | 0.98 | 0.96 | 0.02 (0.01-0.03) | 23.37 (7) | 0.99 | 0.97 | 0.02 (0.01-0.03) |
| Partially adjusted model | 36.71 (17) | 0.99 | 0.97 | 0.01 (0.01-0.02) | 37.44 (17) | 0.99 | 0.98 | 0.01 (0.01-0.02) |
| Fully adjusted model | 41.8 (21) | 0.99 | 0.98 | 0.01 (0.01-0.02) | 35.56 (21) | 1.0 | 0.99 | 0.10 (0.00-0.02) |
| **Nights in nursing home** |  |  |  |  |  |  |  |  |
| Unadjusted model | 13.29 (7) | 1.0 | 1.0 | 0.01 (0.00-0.02) | 27.12 (7) | 1.0 | 0.99 | 0.02 (0.01-0.03) |
| Partially adjusted model | 35.45 (17) | 1.0 | 0.99 | 0.01 (0.01-0.02) | 55.16 (17) | 1.0 | 0.99 | 0.02 (0.01-0.02) |
| Fully adjusted model | 43.0 (21) | 1.0 | 0.99 | 0.01 (0.01-0.02) | 52.37 (21) | 1.0 | 0.99 | 0.02 (0.01-0.02) |

*Note.* Model fit for Table H. Partially adjusted models adjusted for baseline age, gender, ethnicity, education and household income. Fully adjusted models additionally adjusted for baseline depression and morbidity.

**Supplementary Table 10.** Associations between loneliness and social isolation trajectories and baseline healthcare utilisation (2-year prior healthcare)

|  | **Inpatient care** |  | **Outpatient care** | **Nursing home care** |
| --- | --- | --- | --- | --- |
|  | hospitalisation | re-hospitalisation | physician visits |  |
| **Loneliness trajectory** | *β (SE)* | *β (SE)* | *β (SE)* | *β (SE)* |
| *I lon* | 0.03 (0.02) | 0.01 (0.02) | -0.02 (0.02) | 0.00 (0.01) |
| *S lon* | 0.02 (0.04) | 0.05 (0.04) | 0.02 (0.04) | 0.02 (0.03) |
| Model fit |  |  |  |  |
| chi2(df) | 19.14 (9) | 18.36 (9) | 18.51 (9) | 20.33 (9) |
| CFI | 1.0 | 1.0 | 1.0 | 1.0 |
| TLI | 0.99 | 0.99 | 0.99 | 0.99 |
| RMSEA (90% CI) | 0.01 (0.01-0.02) | 0.01 (0.00-0.02) | 0.01 (0.00-0.02) | 0.01 (0.01-0.02) |
| **Social isolation trajectory** | *β (SE)* | *β (SE)* | *β (SE)* | *β (SE)* |
| *I iso* | -0.03 (0.02) | -0.001 (0.02) | -0.04 (0.02) * | 0.03 (0.01) * |
| *S iso* | 0.02 (0.02) | 0.02 (0.02) | -0.03 (0.02) | -0.02 (0.02) |
| Model fit |  |  |  |  |
| chi2(df) | 14.09 (9) | 15.26 (9) | 12.71 (9) | 14.37 (9) |
| CFI | 1.0 | 1.0 | 1.0 | 1.0 |
| TLI | 1.0 | 1.0 | 1.0 | 1.0 |
| RMSEA (90% CI) | 0.01 (0.01-0.02) | 0.01 (0.00-0.02) | 0.01 (0.00-0.02) | 0.01 (0.00-0.02) |

*Note.* *p < 0.05, **p < 0.01, ***p < 0.001. β, Standardised estimates. We applied a cut-off of scores ≥3 for loneliness and a cut-off of ≥6 for social isolation in the estimates. I lon, intercept of loneliness; I care, intercept of healthcare utilisation; S lon, slope of loneliness; S care, slope of healthcare utilisation. Single-headed arrows represent regression effects. All models adjusted for baseline age, gender, ethnicity, education, household income, depression and morbidity.

**Supplementary Table 11.** Longitudinal associations between baseline loneliness and social isolation (including interaction term) and healthcare utilisation (2-year prior healthcare) trajectories

|  | **Inpatient care** |  | **Outpatient care** | **Nursing home care** |
| --- | --- | --- | --- | --- |
|  | hospital stays | re-admission to hospital | physician visits | nursing home stays |
| **loneliness & social isolation** | *β (SE)* | *β (SE)* | *β (SE)* | *β (SE)* |
| loneliness I care | NC | 0.01 (0.80) | -0.01 (4.12) | NC |
| loneliness S care | NC | 0.08 (0.13) | -0.02 (0.60) | NC |
| social isolation I care | NC | 0.02 (2.50) | -0.30 (10.24) | NC |
| social isolation S care | NC | 0.02 (0.71) | -0.09 (0.31) | NC |
| Iso* lon I care | NC | -0.13 (35.9) | 0.18 (462.24) | NC |
| Iso* lon S care | NC | -0.04 (3.23) | 0.02 (43.5) | NC |
| Model fit |  |  |  |  |
| chi2(df) | NC | 15.07 (9) | 3.90 (9) | NC |
| CFI | NC | 0.94 | 1.0 | NC |
| TLI | NC | 0.79 | 1.0 | NC |
| RMSEA (90% CI) | NC | 0.01 (0.00-0.03) | 0.00 (0.00-0.01) | NC |

*Note.* *p < 0.05, **p < 0.01, ***p < 0.001. β, Standardised estimates. We applied a cut-off of scores ≥3 for loneliness and a cut-off of ≥6 for social isolation in the estimates. I lon, intercept of loneliness; I care, intercept of healthcare utilisation; S lon, slope of loneliness; S care, slope of healthcare utilisation. Single-headed arrows represent regression effects; Models adjusted for baseline age, gender, ethnicity, education, household income, and additionally adjusted for baseline depression and morbidity.

**Supplementary Table 12.** Baseline characteristics of samples at baseline and longitudinal sample

| Characteristics | Wave 1  (n=13,830) | Longitudinal analyses sample  (n=6,832) |
| --- | --- | --- |
| Baseline age (Mean, SD) | 66.7 (10.3) | 63.7 (8.0) |
| ≤59 | 31.5% | 38.5% |
| 60-69 | 33.1% | 37.9% |
| 70-79 | 21.1% | 19.1% |
| 80 and above | 14.4% | 4.5% |
| Household income (quartiles) |  |  |
| <$19,000 | 22.1% | 15.4% |
| $19,000-$39,999 | 24.5% | 22.0% |
| $40,000-$79,999 | 26.7% | 29.9% |
| ≥$80,000 | 26.7% | 32.7% |
| Chronic illnesses |  |  |
| No morbidity | 42.1% | 49.2% |
| morbidity | 57.9% | 50.8% |
| Depression |  |  |
| No depression | 78.4% | 83.3% |
| Depression | 21.6% | 16.7% |
